# MiR-186 suppresses the growth and metastasis of bladder cancer by targeting NSBP1

**DOI:** 10.1186/s13000-015-0372-3

**Published:** 2015-08-20

**Authors:** Kun Yao, Leye He, Yu Gan, Qing Zeng, Yingbo Dai, Jing Tan

**Affiliations:** Department of Urology, The Third Xiangya Hospital of Central South University, 138 Tongzipo Road, Changsha, 410013 Hunan China

## Abstract

**Background:**

Increasing evidence has shown that microRNAs function as oncogenes or tumor suppressors in human malignancies, but the roles of miR-186 in human bladder cancer (BC) is still unclear.

**Methods:**

First, quantitative real-time PCR (qRT-PCR) was performed to detect miR-186 expression in bladder cancer tissues and cell lines. Then, Bioinformatics analysis, combined with luciferase reporter assay demonstrated the target gene of miR-186. Finally, the roles of miR-186 in regulation of tumor proliferation and invasion were further investigated.

**Results:**

Here, our study showed miR-186 was down-regulated in bladder cancer tissues and cell lines. Luciferase reporter assay showed that miR-186 targets NSBP1 3′-untranslated region (UTR) directly and suppresses NSBP1 (HMGN5) expression in human bladder cancer cells. NSBP1 siRNA- and miR-186-mediated NSBP1 knock-down experiments revealed that miR-186 suppresses cell proliferation and invasion through suppression of NSBP1 expression. Expression analysis of a set of epithelial-mesenchymal transition (EMT) markers showed that NSBP1 involves miR-186 suppressed EMT which reducing the expression of mesenchymal markers (vimentin and N-cadherin) and inducing the expression of epithelial marker (E-cadherin).

**Conclusions:**

Our data first time identified miR-186 as the upstream regulator of NSBP1 and also suggest miR-186-suppressed NSBP1 as a novel therapeutic approach for bladder cancer.

## Background

Bladder cancer is the most common malignancy involving the urinary system with more than 350,000 new cases diagnosed globally each year [1, 2]. Bladder cancer is the fourth most common cancer in males and ninth most common in females, and is by far the most frequent urological malignancy in China [3]. Despite significant advances in accurate and effective diagnostic and therapeutic methods, bladder cancer remains a highly prevalent and lethal malignancy [4]. Therefore, it is urgent for novel treatment strategies based on new molecular networks to improve the poor prognosis in patients with bladder cancer.

High mobility group N (HMGN) proteins are a family of ubiquitous nuclear proteins which modify the structure of chromatin to attain a conformation that facilitates and enhances transcription, histone modifications, replication and repair [5, 6]. NSBP1 (Nucleosomal Binding Protein 1), also named HMGN5, is a new member of the HMGN protein family, is reported to bind to the nucleosomes via nucleosomal binding domain (NBD), unfold chromatin, and modulate gene transcription [7]. Accumulating studies showed that NSBP1 was abundantly expressed in various types of cancer, including gliomas [8], clear cell renal cell carcinoma [9] and prostate cancer [10]. Recently, Wahafu et al. revealed that NSBP1 is highly expressed in human bladder cancer and promotes the proliferation and invasion of bladder cancer cells [11]. However, it is not known whether NSBP1 expression is regulated by specific miRNAs in bladder cancer.

MicroRNA (miRNA), an abundant group of endogenous non-coding single strand RNAs of 22 nucleotides, participate in the regulation of a range of biological processes including cell proliferation, apoptosis, invasion, migration, differentiation, by regulating the expression of genes at post-transcriptional level [12–15]. Increasing evidence indicates that the miRNAs, function as either oncogenes or tumor suppressors, are aberrantly expressed and contribute to cancer progression as a result of changes in expression of their target genes in various cancers such as breast cancer, lung cancer, pancreatic cancer and nasopharyngeal carcinoma [16–21]. Accumulating studies showed that the deregulated expression of miR-186 was observed in various cancers. For example, miR-186 was reported to be significantly upregulated in most pancreatic cancer [22]. Recently, miR-186 function as a tumor suppressive miRNA and miR-186 expression level is down-regulated in various human malignancies: endometrial cancer [23], prostate cancer [24], medulloblastomas [25], non-small cell lung carcinoma [26, 27]. However, the expression and mechanism of miR-186 in bladder cancer remain unclear.

In this study, we detected that miR‑186 is significantly downregulated in bladder cancer cell lines. NSBP1 is a direct target of miR-186 and the overexpression of miR-186 suppresses cell proliferation and invasion of bladder cancer through suppression of NSBP1 expression and EMT.

## Methods

### Human tissue specimens

Twenty clinical BC tissues and their corresponding noncancerous bladder tissues used in this study were obtained from The Third Xiangya Hospital (Changsha, China) after surgical resection. All samples were immediately snapped frozen in liquid nitrogen and stored at −80 °C until RNA extraction. Informed consents were obtained from each patient to approve the use of their tissues for research purposes. The study protocol was approved by the Institute Research Ethics Committee at Central South University.

### Cell culture and transfection

The human bladder cancer cell lines (J82, HT1376, RT4, T24 and TCCSUP) and immortalized human bladder epithelium (HCV29) cells were cultured in DMEM (Invitrogen) supplemented with 10 % FCS at 37 °C in 5 % CO_2_ cell culture incubator. miR-186 mimics and scramble control mimics (GenePharma, Suzhou, China) were transfected in J82 cells at a final concentration of 50 nM using Lipofectamine 2000 reagent (Invitrogen).

### qRT-PCR

Total RNA was isolated from tissues and cell lines using the miRNeasy Mini Kit (Qiagen). The miRNA Q-PCR Detection Kit (GeneCopoeia) was used for quantification of miRNA levels according to the manufacturer’s protocol. The protocol was conducted for 35 cycles at 95 °C for 3 min, 95 °C for 12 s, and 58 °C for 30 s. The PCR amplification for the quantification of the miR-186 and U6 was performed using TaqMan miRNA Reverse Transcription Kit (Applied Biosystems, Foster City, CA, USA) and TaqMan Human MiRNA Assay Kit (Applied Biosystems, Foster City, CA, USA). The relative expression of miR-186 was shown as fold difference relative to U6. The PCR amplification for the quantification of the NSBP1 and GAPDH mRNAs was performed using an ABI PRISM 7300 Sequence Detection System (Applied Biosystems, Foster City, CA, USA) and a SYBR®Premix Ex Taq™ ii (Perfect Real Time) Kit (Takara Bio, Shiga, Japan). The primers were as follows: miR-186: 5′- GCGGCGCAAAGAATTCTCCT-3′; miR-186 mimics, forward primer: 5′-GCGGCGCAAAGAATTCTCCT-3′ and reverse primer: 5′-GTGCAGGGTCCGAGGT-3′; NSBP1, forward primer: 5′-TCGGCTTTTTTTCTGCTGACTAA-3 and reverse primer: 5′-CTCTTTGGCTCCTGCCTCAT-3′. β-actin, forward primer: 5′- CATTAAGGAGAAGCTGTGCT-3′ and reverse primer: 5′- GTTGAAGGTAGTTTCGTGGA -3′.

### Western blot

Whole cell extracts were prepared with a cell lysis reagent (Sigma-Aldrich, St. Louis, MO, USA) according to the manual, and then, the protein was quantified by a BCA assay (Pierce, Rockford, IL, USA). Then, the protein samples were separated by SDS-PAGE (10 %) and detected by Western blot using polyclonal (rabbit) anti- NSBP1, anti-E-cadherin, anti-N-cadherin and anti-Vimentin antibody (Santa Cruz Bio-technology, Santa Cruz, CA, USA). Goat anti-rabbit IgG (Pierce, Rockford, IL, USA) secondary antibody conjugated to horseradish peroxidase and ECL detection systems (SuperSignal West Femto, Pierce) were used for detection.

### Luciferase reporter assay

The 3′-UTR sequence of NSBP1 was amplified from normal human genomic DNA and subcloned into the pmirGLO luciferase reporter vector (Promega). HEK 293 T cells (3.5 × 10 ^4^) were seeded in triplicate in 24-well plates and cotransfected with wild-type (WT) or mutant (Mut) 3′-UTR vectors and miR-186 mimics using Lipofectamine 2000. After 48 h, the cells were assayed for luciferase activity using the Dual-Luciferase Reporter Assay System (Promega) by following the manufacturer’s instructions. The firefly luciferase activities were normalized to Renilla luciferase activity. The firefly luciferase activity of the cells that were transfected with miRNA mimics or inhibitors is represented as the percentage of activity relative to that of cells that were transfected with negative controls. All experiments were performed in triplicate.

### Cell proliferation assay and invasion assay

The 3-(4,5-dimethylthiazal-2-yl)-2,5-diphenyl-tetrazolium bromide (MTT) assay was used to estimate cell viability [28]. Briefly, cells were plated at a density of 1 × 10 ^4^ cells per well in 96-well plates. After exposure to specific treatment, the cells were incubated with MTT at a final concentration of 0.5 mg/ml for 4 h at 37 °C. After the removal of the medium, 150 mM DMSO solutions were added to dissolve the formazan crystals. The absorbance was read at 570 nm using a multi-well scanning spectrophotometer reader. Cells in the control group were considered 100 % viable.

The capability of cell invasion was examined by transwell invasion assay. Cells were cultivated to 80 % confluence on the 12-well plates. Then, we observed the procedures of cellular growth at 72 h. All the experiments were repeated in triplicate. The transwell migration chambers were used to evaluate cell invasion. Then invasing cells across the membrane were counted under a light microscope.

### Statistical analysis

Each experiment was repeated at least three times. Data were shown as mean ± s.d and analyzed using SPSS 18.0. Statistical comparisons between groups were analyzed using correlation between expression levels of miR-186 and its target genes in BC tissues was analyzed using Spearman’s correlation coefficient. Student’s *t*-test and a two-tailed *p* < 0.05 were considered to indicate statistical significance.

## Results

### The expression of miR-186 and NSBP1 in BC tissues and cell lines

**Fig. 1:**
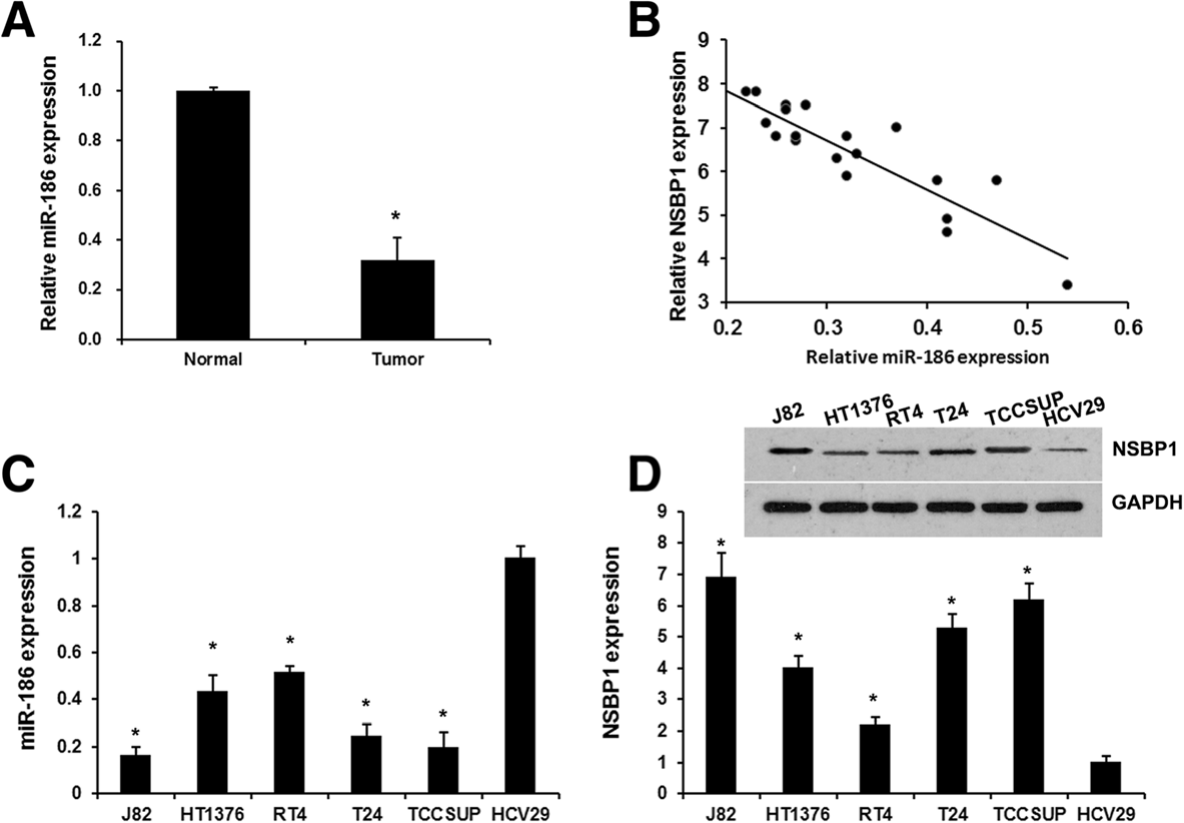
The expression of miR-186 and NSBP1 in bladder cancer tissues and cell lines. **a**, miR-186 expression is markedly decreased in tumor samples compared to adjacent noncancerous bladder tissues. **b** The inverse correlation between NSBP1 and miR-186 expression in 20 BC samples was determined using Spearman’s correlation analysis (*r* = −0.8946, *P* <0.05) * *P* <0.05. **c**, qRT-PCR analysis revealed the miR-186 expression in human bladder cancer cell lines (J82, HT1376, RT4, T24 and TCCSUP) and immortalized human bladder epithelium (HCV29) cells. **d**, Western blot analysis revealed the NSBP1 expression in human bladder cancer cell lines (J82, HT1376, RT4, T24 and TCCSUP) and immortalized human bladder epithelium (HCV29) cells. Each bar represents the mean of three independent experiments. * *P* < 0.01 versus HCV29 cell line

Recently, miR-186 function as a tumor suppressive miRNA and miR-186 expression level is down-regulated in various human malignancies [26, 27]. However, the expression and mechanism of miR-186 in bladder cancer remain unclear. Here, we analyzed the miR-186 expression in 20 paired clinical BC and adjacent noncancerous bladder tissues using qRT-PCR. We found that miR-186 expression was significantly decreased in BC tissues relative to the matched non-tumor tissues (*P* < 0.05, Fig. 1a). Moreover, our data indicated that there was an inverse correlation between miR-186 and NSBP1 expression (Fig. 1b).

Next, we employed qRT-PCR to detect miR-186 levels in human bladder cancer cell lines (J82, HT1376, RT4, T24 and TCCSUP) and immortalized human bladder epithelium (HCV29) cells. The miR-186 expression was downregulated in all bladder cancer cell lines as compared with that in HCV29 (Fig. 1c). These data indicates that miR-186 may function as tumor suppressor in bladder cancer cells.

We next assayed the NSBP1 expression levels in human bladder cancer cell lines (J82, HT1376, RT4, T24 and TCCSUP) and immortalized human bladder epithelium (HCV29) cells. Consistent with previous studies [11], the NSBP1 expression was upregulated in all BC cell lines as compared with that in HCV29 (Fig. 1d). These data indicates that NSBP1 function as oncogene in bladder cancer cells.

### miR-186 directly targeted NSBP1

**Fig. 2:**
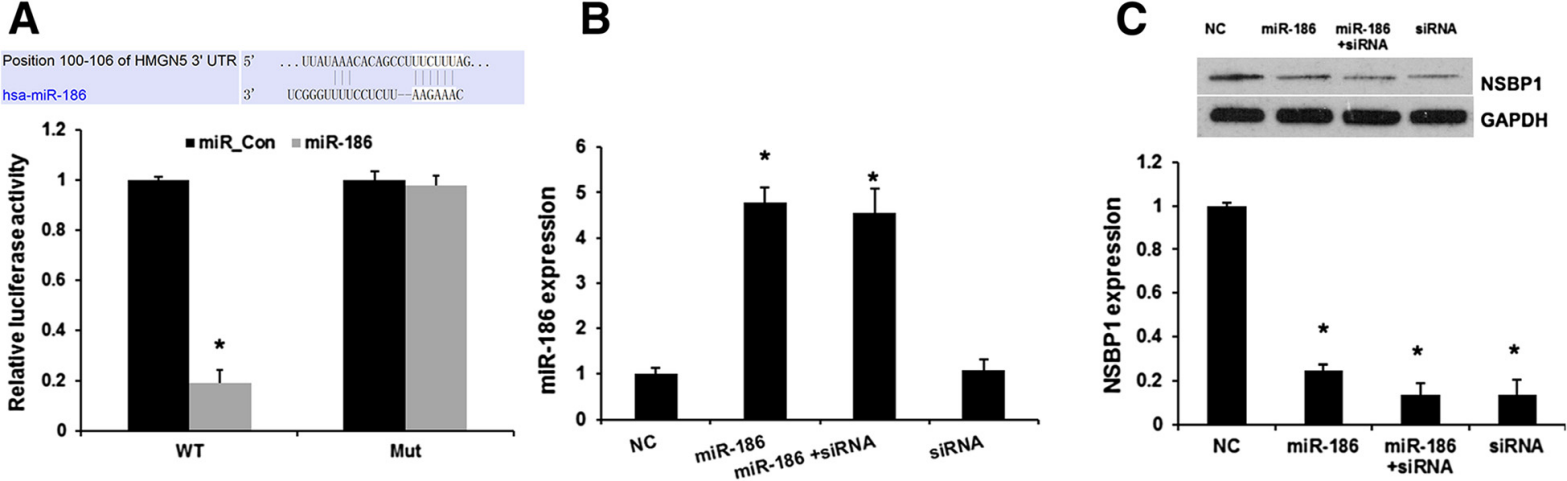
miR-186 directly targeted NSBP1. **a**, Sequence alignment of miR-186 and 3′ UTR of NSBP1 using mirco-RNA.org. Luciferase reporter assay with co-transfection of wild-type or mutant NSBP1 and miR-186 mimics or miR–control (scramble control mimics).in HEK 293 T cells. Error bars represent ± S.E. and *, *p* < 0.01 versus negative control (scramble control mimics). **b**, qRT-PCR analysis revealed the effects of NSBP1 siRNA and miR-186 mimics on the expression level of miR-186. **c**, Western blot analysis revealed the effects of NSBP1 siRNA and miR-186 mimics on the expression level of NSBP1. Error bars represent ± S.E. and *, *p* < 0.01 versus NC (scramble control siRNA)

In order to elucidate the underlying molecular mechanism, we performed a bioinformatic analysis using mirco-RNA.org (http://www.microrna.org/microrna/home.do) to predict the possible target gene of miR-186. We found that NSBP1 contained theoretical miR-186 binding sites in its 3′ UTR (Fig. 2a). To further confirm these results, we constructed luciferase reporter vectors containing the wild-type (Wt) or mutant (Mut) miR-186 target sequences of the NSBP1 3′-UTR (Fig. 2a). Overexpression of miR-186 significantly inhibited the luciferase activity of the Wt NSBP1 3′-UTR reporter gene but not the Mut reporter gene (Fig. 2a).

Next, we transfected NSBP1 siRNAs in J82 cells (Fig. 2b). Western blot analysis revealed NSBP1 expression were significantly decreased by NSBP1 siRNA, compared with negative control group (scramble control siRNA) (*P* < 0.05). In addition, overexpression of miR-186 markedly reduced the expression of NSBP1 (Fig. 2b), but silenced NSBP1 did not affect miR-186 expression. These results demonstrated that NSBP1 is a direct target of miR-186 in BC cells (Fig. 2c).

### miR-186 regulated BC cell proliferation and invasion by suppressing NSBP1 expression

**Fig. 3:**
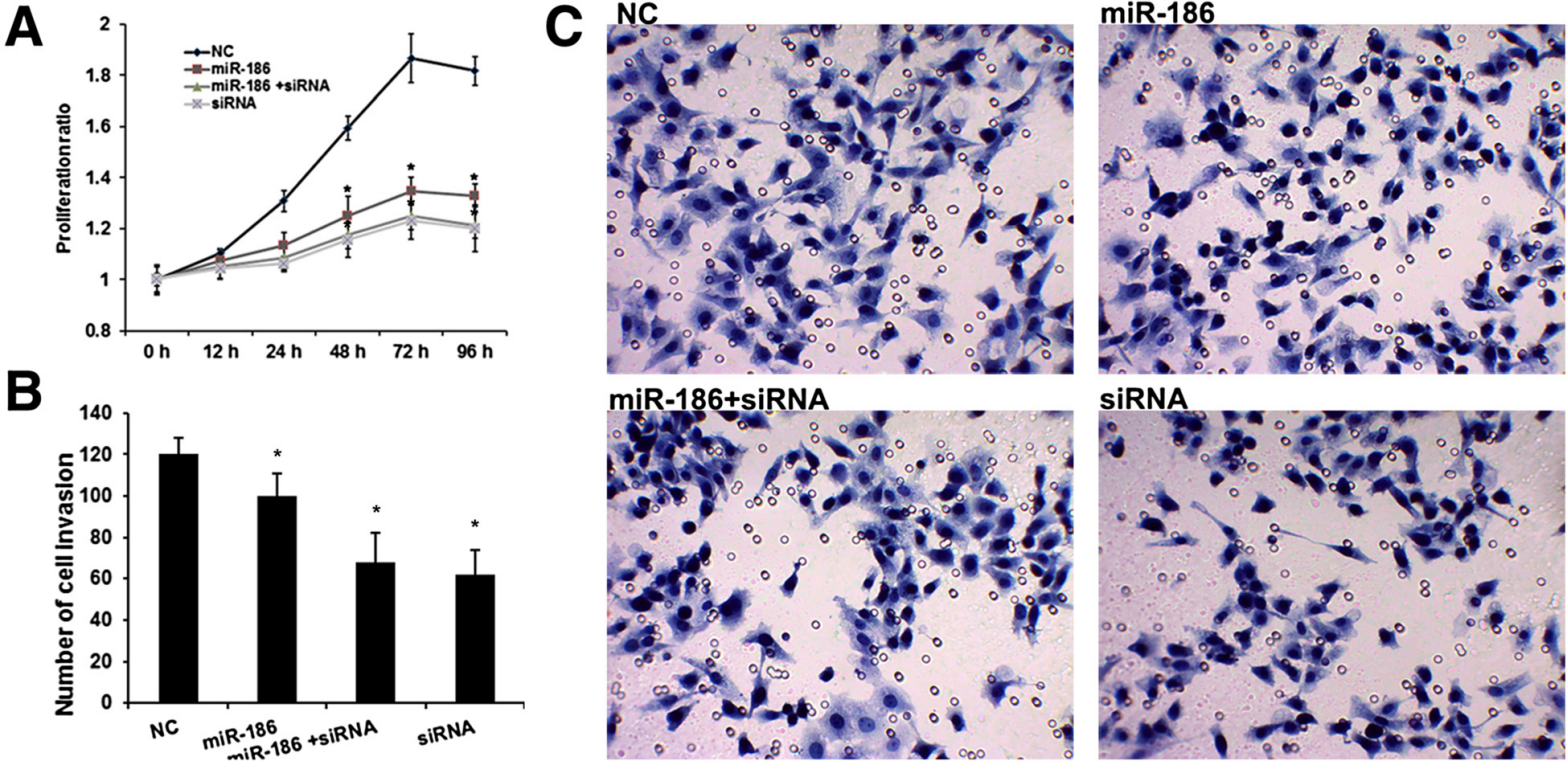
miR-186 regulates bladder cancer cell proliferation and invasion by suppressing NSBP1 expression. **a**, Overexpressed miR-186 and silenced NSBP1 suppressed cell proliferation. Error bars represent ± S.E. and *, *p* < 0.01 versus NC (scramble control siRNA). **b** and **c**, Overexpressed miR-186 and silenced NSBP1 suppressed cell invasion. Error bars represent ± S.E. and *, *p* < 0.05 versus NC (scramble control siRNA)

To determine the role of NSBP1 and miR-186 in the bladder cancer cell growth and metastasis, J82 cells were transiently co-transfected with **NSBP1** siRNA (siRNA) and miR-186 mimic (miR-186). Consistent with the effects induced by overexpression of miR-186, knockdown of NSBP1 significantly suppressed the cell viability (Fig. 3a) and invasion (Fig. 3b and c), whereas overexpression of miR-186 did not have further suppressive effects on cell growth and metastasis in NSBP1-siRNA -transfected J82 cells.

### MiR-186 induces EMT of BC cells by suppressing NSBP1 expression

**Fig. 4:**
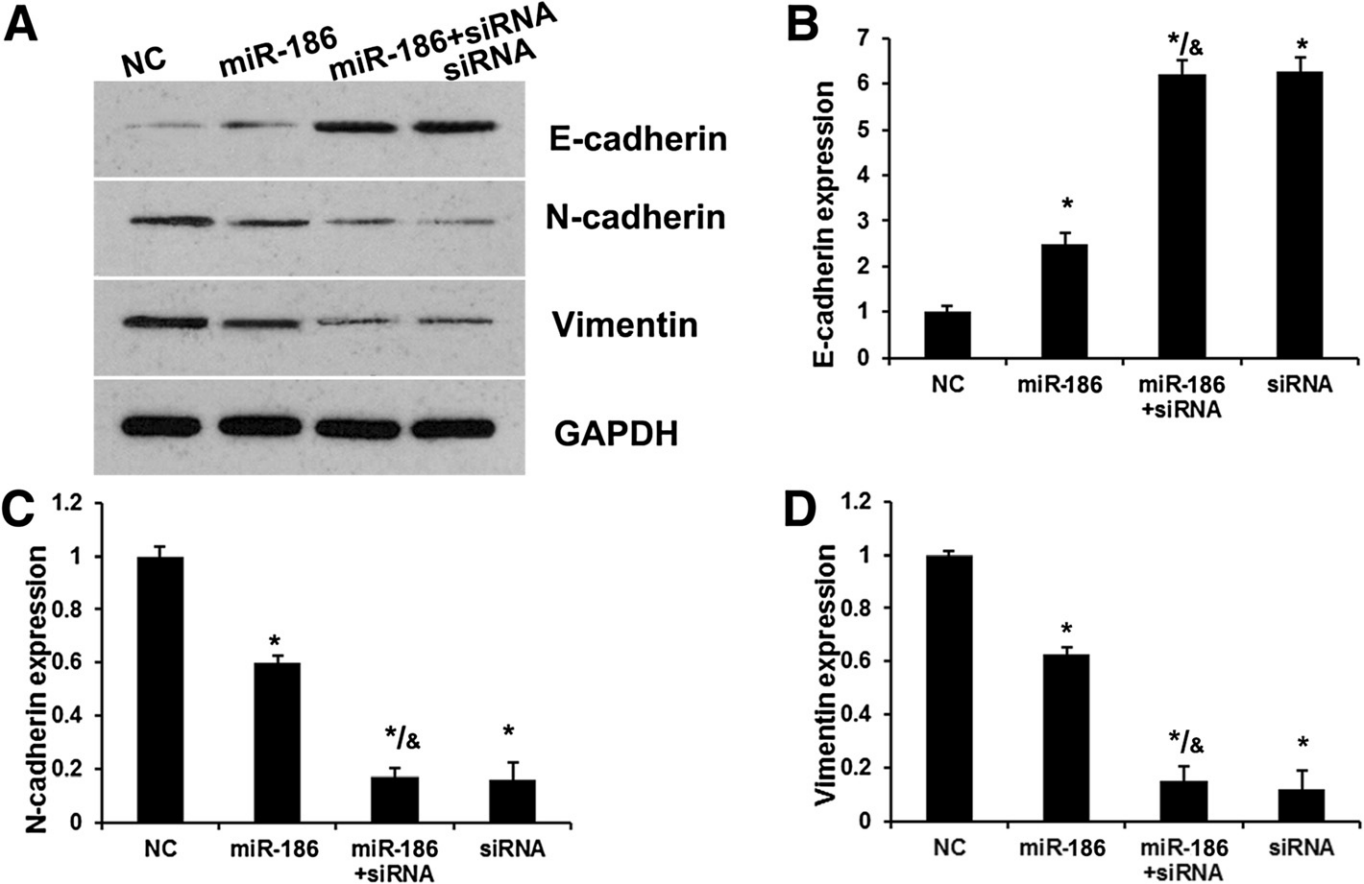
MiR-186 induces EMT of BC cells by suppressing NSBP1 expression. **a**, Western bolt analysis revealed the effects of miR-186 and NSBP1 on EMT-relative protein expression. **b**, The effects of miR-186 and NSBP1 on E-cadherin expression. **c**, The effects of miR-186 and NSBP1 on N-cadherin expression. **d**, The effects of miR-186 and NSBP1 on Vimentin expression. Error bars represent ± S.E. and *, *p* < 0.01 versus NC (scramble control siRNA). &, *p* < 0.01 versus miR-186 group

EMT has been identified as a key role in the invasion of various cancer cells by the transformation of polarized and adherent epithelial cells into motile and invasive mesenchymal cells. Here, to explore protein regulated by miR-186 in the EMT process, we investigated the expression of three EMT related proteins, E-cadherin, N-cadherin and Vimentin by Western blot. Bladder cancer cells were transfected with NC, miR-186 mimics, NSBP1 siRNA and co-transfected with miR-186 mimics and NSBP1 siRNA. Results indicated the expression of E-cadherin was increased in miR-186 mimics group compared with NC (Fig. 4a and b). Moreover, E-cadherin expression in siRNA group was higher than that in miR-186 group (miR-186 mimics) and similar with that in miR-186 + siRNA group. N-cadherin and Vimentin was downregulated significantly in miR-186 group (Fig. 4a, c and d). Moreover, N-cadherin and Vimentin expression in siRNA group were lower than that in miR-186 group (miR-186 mimics) and similar with that in miR-186 + siRNA group. This indicated miR-186 represses the expression of N-cadherin and Vimentin, while promoting the induction of E-cadherin by targeting NSBP1.

## Discussion

NSBP1, also named HMGN5, is a novel member of HMGN family, which modifies the structure of chromatin to attain a conformation that facilitates and enhances transcription, histone modifications, replication and repair [5, 6]. Elevated NSBP1 expression is found in a number of tumors [8, 11], and downregulation of the NSBP1 gene can inhibit the tumor cell proliferation *in vitro* and *in vivo* [10]. Therefore, NSBP1 inhibition has recently emerged as a potential target of drug therapy. Increased NSBP1 expression has been reported in bladder cancer tissues and cell lines [11]. However, the regulation of NSBP1 by miRNAs in bladder cancer has not been explored in detail.

In this study, we examined both the regulation of the NSBP1 pathway by miR-186 in bladder cancer, as well as its functional significance. The miR-186 has been commonly deregulated in various cancers. For example, miR-186 was reported to be significantly upregulated in most pancreatic cancer [22]. Recently, miR-186 function as a tumor suppressive miRNA and miR-186 expression level is down-regulated in endometrial cancer [23], prostate cancer [24], medulloblastomas [25], non-small cell lung carcinoma [26, 27]. However, little is known of its expression and potential function in bladder cancer. Here, we report downregulation of miR-186 and demonstrate its role as a tumor suppressor in bladder cancer. We observed miR-186 to be downregulated in bladder cancer cell lines compared with bladder epithelium (HCV29) cells. The miRNA target prediction websites www.microRNA.org and TargetScan identified the NSBP1 as a possible target of miR-186. Our results demonstrated that miR-186 directly targets the 3′UTR of NSBP1, as its overexpression was associated with suppression of luciferase activity in a reporter plasmid driven by the NSBP1 -3′UTR.

In addition, a significant downregulation of NSBP1 protein levels was observed following miR-186 overexpression, indicating the post-transcriptional regulation of NSBP1 via targeting its 3′UTR. NSBP1 expression is frequently elevated in a variety of cancers, including gliomas, prostate cancer and clear cell renal cell carcinoma as well as bladder cancer [8–11], and may therefore represent a promising molecular target for anticancer therapy. However, there are no reports about specific miRNAs regulating NSBP1 expression in tumors. NSBP1 has been reported to correlated with the increased tumor grade and pathologic stage, and lymph node metastasis in bladder cancer [11], suggesting a role for NSBP1 in the development and/or progression of bladder cancer. Our results suggest silencing of miR-186 as a possible mechanism for NSBP1 overexpression in bladder cancer.

NSBP1 has been reported to overexpressed in human bladder cancer tissues compared with paraneoplastic bladder tissues [11]. Consistent with previous study, we observed NSBP1 to be overexpressed in bladder cancer cell lines compared with bladder epithelium (HCV29) cells. Additional studies identified NSBP1 as a direct functional target of miR-186. miR-186-mediated suppression of NSBP1 attenuates cell proliferation and invasion of bladder cancer. So we speculated that miR-186 function as tumor suppressor and inhibit bladder cancer proliferation and invasion by suppressing NSBP1 expression.

EMT, a dynamic and reversible cellular process, is characterized by loss of cell polarity and intracellular junctions and acquirement of mesenchymal features, which contributes to tumor development and metastasis [29]. Here, we determined the expression of epithelial marker, E-cadherin, and mesenchymal marker, vimentin and N-cadherin in bladder cancer cells with altering expression of miR-186 and NSBP1. Interestingly, we demonstrated that miR-186 mimics and NSBP1 siRNA inhibited EMT and were associated with reduced expression of E-cadherin and elevated expression of N-cadherin and vimentin in bladder cancer. Taken together, miR-186 suppress BC cell EMT by targeting NSBP1.

## Conclusion

In conclusion, our study demonstrates that miR-186 is significantly downregulated in bladder cancer. Ectopic miR-186 results in suppression of the proliferative, invasive ability and EMT of bladder cancer by directly targeting NSBP1. Overall, these studies describe a promising therapeutic role for miR-186 in bladder cancer, which appears to act at least in part by mimicking pharmacological inhibitors of NSBP1.
